# S100A12 as diagnostic tool in the differential diagnosis of sJIA associated MAS vs. hereditary or acquired HLH

**DOI:** 10.1186/1546-0096-13-S1-O64

**Published:** 2015-09-28

**Authors:** D Holzinger, N Fall, A Grom, W de Jager, S Vastert, R Strippoli, C Bracaglia, E Sundberg, A Horne, S Ehl, F De Benedetti, K Beutel, D Foell

**Affiliations:** 1 University Children’s Hospital Muenster, Department of Pediatric Rheumatology and Immunology, Muenster, Germany; 2 Cincinnati Children's Hospital Medical Center, Divisions of Rheumatology, Cincinnati, USA; 3 University Medical Center Utrecht, Laboratory of Translational Immunolgy, Utrecht, Netherlands; 4 University Medical Center Utrecht, Department of Pediatric Rheumatology and Immunology, Utrecht, Netherlands; 5 Sapienza University of Rome, Department of Cellular Biotechnology and Hematology, Rome, Italy; 6 IRCCS Ospedale Pediatrico Bambino Gesù, UO Reumatologia, Rome, Italy; 7 Karolinska University Hospital Solna, Paediatric Rheumatology Unit, Stockholm, Sweden; 8 Karolinska University Hospital Solna, Childhood Cancer Research Unit, Stockholm, Sweden; 9 University Hospital Freiburg, CCI - Center for Chronic Immunodeficiency, Freiburg, Germany; 10 Children's Hospital, Technische Universität München, Department of Pediatric Hematology and Oncology, Munich, Germany

## Question

Macrophage activation syndrome is a severe complication of autoimmune and autoinflammatory disease. MAS is most strongly associated with systemic juvenile idiopathic arthritis (sJIA) but can also be seen in Kawasaki disease, SLE or IBD. Clinically, MAS is strikingly similar to hemophagocytic lymphohistiocytosis (HLH) and the initial differentiation between sJIA-associated MAS and hereditary HLH or acquired HLH is very difficult. Due to recent advances in the description of HLH-related gene defects, most patients with hereditary HLH can be identified through genetic or functional analysis (intracellular perforin, SLAM-associated protein analysis, x-linked inhibitor of apoptosis, CD107 degranulation assay). Unfortunately these investigations are not always easily available. Since viral infections such as EBV and CMV can trigger both hereditary and acquired forms of HLH, the presence of a viral trigger in a patient with HLH does not necessarily allow classification of the disease as acquired HLH.

S100A12 is an endogenous TLR4 ligand that induces monocyte activation, thereby acting as an amplifier of innate immunity during early inflammation. S100A12 is highly overexpressed in sJIA, and the assessment of S100A12 serum levels helps distinguish sJIA from other febrile illnesses. The main goal of this study was to determine whether S100A12 might help distinguish sJIA-associated MAS from HLH.

## Methods

S100A12 serum levels were assessed in 177 samples obtained from 114 unique patients using the in-house ELISA kit. Of 177 samples, 152 samples were also available for a multiplex immunoassay including 53 cytokines and chemokines. Serum samples were obtained from 9 healthy controls, sJIA patients without MAS (17 active/ 19 remission), sJIA patients with MAS (33 active/ 33 remission), acquired HLH (22 active/ 20 remission) and 33 patients with hereditary HLH at disease onset. Additional data obtained at the time of serum collection included clinical features, conventional laboratory markers (CRP, ESR, differential blood count, fibrinogen, ferritin, triglycerides) and when available, NK cell function test results and sCD25 levels.

## Results

Patients with hereditary and acquired HLH could be differentiated by S100A12 serum levels from patients with sJIA-associated MAS. S100A12 levels >1400 ng/ml were seen only in patients with active sJIA (with MAS (mean±SD 5470±3042 ng/m)l or without MAS (4150±3251 ng/ml), but not in patients with acquired (451±351 ng/ml) or hereditary HLH (216±170 ng/ml). Healthy controls were in the range of 85±44 ng/ml. Although S100A12 levels correlated closely with disease activity in sJIA patients as determined by JIA core set criteria, there was no significant difference between sJIA patients with or without MAS.

## Conclusions

S100A12 serum levels are useful to differentiate between sJIA-associated MAS and inherited or acquired HLH. The combination with conventional laboratory markers, serum cytokine profiles and clinical characteristics might allow creating a diagnostic panel for the differentiation of MAS vs. HLH. Since at the onset of disease sJIA MAS and acquired HLH are difficult to discriminate this might be a helpful diagnostic tool.

